# A two-dimensional framework for profiling online reviewer behavior

**DOI:** 10.1371/journal.pone.0344988

**Published:** 2026-03-25

**Authors:** Luisa Stracqualursi, Patrizia Agati

**Affiliations:** Department of Statistics, University of Bologna, Bologna, Italy; Max Rubner-Institut the Federal Research Institute of Nutrition and Food: Max Rubner-Institut Bundesforschungsinstitut fur Ernahrung und Lebensmittel, GERMANY

## Abstract

Consumers frequently rely on extreme online reviews—highly positive or highly negative—for clarity and detailed insights. However, conflicting extremes can generate confusion and erode trust in rating systems, highlighting the need for additional metrics that provide deeper insight into reviewer behavior. To address this, we introduce a novel and intuitive two-dimensional framework for profiling reviewer behavior through two complementary indices: the Reviewer Extremeness Index (REI), which quantifies the frequency of extreme ratings, and the Reviewer Polarity Index (RPI), which measures the directional imbalance between positive and negative extremes, along with its intensity. The framework maps each reviewer onto a two-dimensional plane whose axes are REI and RPI, identifying nine archetypal profiles of reviewers’ historical extreme behaviors. As a case study, we applied this approach to three million Amazon book reviews, demonstrating its practical value in a real-world context. This framework provides dual utility. For consumers, it offers crucial contextual information: knowing a reviewer’s archetype allows for a more nuanced interpretation of their feedback. For online retail platforms, the framework serves as a scalable tool to monitor reviewer behavior and identify systematic rating patterns that may warrant further scrutiny, such as those potentially associated with incentivized reviewing. By making reviewer tendencies transparent, our model contributes to a more reliable and trustworthy digital marketplace ecosystem.

## 1 Introduction

In today’s digital age, e-commerce platforms heavily rely on user-generated content (UGC) to assist consumers in making informed purchasing decisions. This UGC typically consists of online user reviews, composed of two primary elements: a star rating (often ranging from 1 to 5 stars) and detailed written comments highlighting various aspects such as quality, usability, and value for money [1].

Online reviews represent a contemporary form of word-of-mouth (WOM) communication, with the advantage of being publicly accessible and reaching extensive audiences. Consequently, reviews significantly influence consumer behavior and product sales, positively through endorsements and negatively through criticisms [2,3].

Nevertheless, the phenomenon of polarized reviews poses significant challenges [4]. While consumers generally seek out extreme ratings in the belief that they offer more explicit insights [5], the presence of conflicting extremes generates ambiguity. Prior research indicates that such inconsistency complicates the decision-making process and undermines users’ trust in the reliability of the reviews [6,7]. Furthermore, ambiguity regarding product quality signals has been shown to negatively impact market outcomes [8]. In addition, the practice of incentivized reviews—where reviewers consistently give overly positive ratings in exchange for benefits—further compromises the authenticity and reliability of online reviews [9–15].

Traditional methods used by platforms to evaluate review quality, such as Amazon’s review helpfulness feedback, are useful but incomplete [1,16]. Indeed, a non-negligible percentage of reviews receive few or no helpful votes, for example those that are recent and have not had sufficient time to accumulate feedback, which reinforces the challenge of accurately gauging the review’s value [17].

Many studies focus on reviewer trustworthiness as a key factor [18] or on strategic behaviors aimed at maximizing visibility [19], which can correlate with extreme rating patterns. Other approaches leveraging machine learning (ML) and natural language processing (NLP) primarily rely on complex textual analyses or network patterns, demanding substantial computational resources and limiting real-time interpretability [20–22].

As mentioned above, several studies highlight the importance of extreme reviews in the consumer decision-making process, as they often emphasize key product features [4,5]. However, shifting the focus from individual reviews to the reviewer’s overall tendency toward extremity enables a deeper understanding: knowing whether a reviewer consistently gives extreme ratings helps contextualize their current opinion on a specific item. This approach is grounded in Source Credibility Theory [23], which posits that the persuasive impact of a message is not intrinsic but heavily dependent on the perceived characteristics of its source. By profiling the reviewer’s historical behavior, we aim to provide the digital equivalent of ‘source knowledge’ [24], reducing the ambiguity of isolated ratings.

For instance, a 5-star rating from a typically critical or balanced reviewer conveys stronger informational value than the same rating from someone who habitually rates positively.

Our proposed framework captures this behavioral tendency and makes it actionable. In this way, it becomes possible—to our knowledge—to obtain a systematic and quantifiable representation of the reviewer’s evaluative profile, introducing an interpretive dimension that has so far been absent from traditional online review systems.

## 2 Related work and theoretical positioning

Building on prior literature, our study is anchored in four key streams. First, research has shown that online ratings follow systematic patterns of polarization, often exhibiting J-shaped distributions [25] and reflecting strong tendencies toward extreme opinions [4]. Second, the perceived helpfulness of reviews depends not only on content but also on rating extremity [1], which highlights the informational value of polarized feedback. Third, recent studies emphasize that reviewer-level characteristics such as trustworthiness and behavioral consistency are central to understanding the reliability of reviews [18,21]. Fourth, our framework operationalizes the principles of Source Credibility Theory in an online environment. While early research identified expertise and trustworthiness as key determinants of persuasion [23,24], the anonymity of digital platforms complicates these assessments. Metzger et al. [26] argue that in the absence of direct personal knowledge, users rely on heuristic cues to evaluate credibility. Recent studies confirm that source characteristics significantly influence travelers’ and consumers’ behavioral intentions [27]. By making the reviewer’s rating history transparent (REI and RPI), our framework provides the necessary cues for consumers to assess the source’s consistency and bias, thereby resolving the ambiguity of mixed product reviews.

To operationalize these theoretical insights and situate our contribution within the methodological landscape, we classify existing approaches into three main categories: (1) unidimensional extremity metrics, (2) clustering-based segmentation, and (3) computational linguistic modeling. Below, we discuss these approaches and highlight how our proposed REI–RPI framework addresses their limitations in providing such transparent cues.

### 2.1 Comparison with existing approaches

To situate our contribution within the existing methodological landscape, we organize the following comparison along three dimensions. For each, we first describe the logic and limitations of the prevailing approach, then explain how the REI–RPI framework addresses them. The label after ‘vs.’ in each subheading identifies the specific property in which our framework improves upon the compared approach. In particular, *bidimensional profiling* refers to the simultaneous capture of both the frequency and the polarity of extreme ratings, which no single metric can achieve. *Deterministic classification* refers to the use of closed-form, rule-based assignment to profiles, as opposed to data-driven model retraining. Finally, *scalability* refers to the ability to operate solely on numerical ratings, without requiring computationally intensive textual analysis.

#### Unidimensional metrics vs. bidimensional profiling.

Unidimensional metrics rely on a single measure to quantify deviations from average behavior. Studies have used variance or entropy to capture dispersion [28], or bias metrics based on deviation from the mean rating [29,30]. **Limitation:** While useful for measuring consistency, single-metric approaches as variance or entropy quantify the dispersion of ratings but do not, on their own, distinguish the direction of the bias. For example, a reviewer who consistently rates 1-star and one who consistently rates 5-stars may show identical variance, yet their impact on the ecosystem is opposite. Comparing a current rating to a reviewer’s personal average can provide a directional signal. However, this operates at the level of a single review, answering the question: “Is this rating higher or lower than usual?” It does not reveal whether the reviewer is systematically positive, negative, or balanced across their entire history. **Our Contribution:** The proposed RPI (Reviewer Polarity Index, eq. 3) captures directionality as a stable, profile-level behavioral trait, classifying each reviewer’s overall tendency toward positive, negative, or balanced extremity. Combined with the REI (eq. 2), this yields a two-dimensional signature that no single metric can provide.

#### Clustering methods vs. deterministic classification.

Clustering-based approaches group reviewers based on behavioral patterns using unsupervised learning [21,31]. **Limitation:** While these methods can reveal meaningful behavioral segments, they require global dataset processing to identify cluster boundaries: the entire corpus must be analyzed, and the model must be retrained whenever new data arrives. Moreover, the resulting clusters often lack transparent interpretation (black box), making it difficult to assign intuitive behavioral labels. **Our Contribution:** The REI–RPI framework applies explicit, closed-form formulas to each reviewer’s individual history. Classification into one of nine profiles follows deterministic rules and does not require training a model on the full reviewer population. It should be noted that, since the NES depends on the observed rating range of each item, the addition of a new review may alter the NES values (eq. 1) of other reviewers who rated the same item. However, this propagation is bounded to the set of co-reviewers of that specific item, and involves only the mechanical reapplication of the same formula—unlike clustering methods, which require a complete re-estimation of the model structure across the entire dataset.

#### NLP approaches vs. scalability.

Computationally complex approaches use large language models (LLMs) to analyze the textual content of reviews [22,32]. **Limitation:** Although powerful, these methods are resource-intensive and language-dependent, limiting their scalability on platforms with millions of users. **Our Contribution:** By relying solely on numerical rating patterns, our framework remains computationally lightweight, language-agnostic, and highly scalable.

As summarized in Table 1, existing approaches tend to focus either on individual reviews or on complex methods that lack immediate transparency. This gap leads to our guiding research question:

*How can reviewer behavior be represented through a transparent and scalable framework that simultaneously captures the frequency and polarity of extreme ratings?*

**Table 1:** Comparison of Reviewer Profiling Approaches. The proposed framework bridges the gap between scalability and interpretability.

Approach Type	Scalability	Interpretability	Captures Extremity?	Captures Direction?	Language Agnostic?
Unidimensional Metrics [28]	High	High	Yes	No	Yes
Clustering Methods [21]	Medium	Low	Yes	Yes	Yes
NLP / LLM Approaches [22]	Low	High	Yes	Yes	No
**Proposed Framework (REI–RPI)**	**High**	**High**	**Yes**	**Yes**	**Yes**

*Note:* The assessments are based on the following criteria. *Scalability* reflects the computational cost of deploying the method across millions of users: approaches requiring model training or textual analysis are rated lower. *Interpretability* refers to how easily the output can be understood without specialized knowledge: methods producing directly readable numerical values (unidimensional metrics, REI–RPI) or human-readable text (NLP) are rated “High,” whereas clustering methods are rated “Low” because cluster membership requires post-hoc labeling to be meaningful. The remaining columns (*Captures Extremity*, *Captures Direction*, *Language Agnostic*) indicate whether each capability is inherently supported by the method (Yes) or not (No).

By addressing this question, we propose the REI–RPI framework as a novel methodological contribution that formalizes reviewer profiling in a two-dimensional space. This dual structure yields a unique behavioral signature for each reviewer, offering a hermeneutic advantage: it digitally mirrors the human heuristic of “knowing your source”, enabling reviews to be interpreted in light of the reviewer’s historical tendencies.

## 3 Methodology

### 3.1 The normalized extremity score (NES)

As a preliminary step, we define the Normalized Extremity Score (NES), a metric computed for each reviewer *j* and item *i*, which quantifies how much an individual rating *s*_*ij*_ deviates from the midpoint of the rating range observed for item *i*.

The midpoint is defined as $\left[(\min(s_{i})+\max(s_{i}))/2\right]$, where $\min(s_{i})$ and $\max(s_{i})$ represent the minimum and maximum ratings received by item *i*, respectively. Accordingly, the resulting formula is:


$$\begin{array}{c} NES_{ij}=\left\{ \begin{array}{cc} 0 & \text{if }\max(s_{i})=\min(s_{i})\\ \frac{s_{ij}-\frac{\min(s_{i})+\max(s_{i})}{2}}{\frac{\max(s_{i})-\min(s_{i})}{2}} & \text{if }\max(s_{i})\neq \min(s_{i}) \end{array}\right.\; \end{array}$$ (1)

*NES* is a normalized score (see proof in S1 Appendix) defined on a signed scale ranging from −1 to 1, where positive and negative values indicate deviations above and below the item’s mid-range rating, respectively.

Notably, NES is a discrete metric whose granularity is determined by the specific rating scale used for each item, such as 1−5 or 1−10, and thus reflects the resolution of the underlying rating system. As an illustrative example, Table 2 shows the behavior of the NES under various combinations of *s*_*ij*_, $\min(s_{i})$, and $\max(s_{i})$, assuming a typical rating scale ranging from 1 to 5.

**Table 2:** NES values for rating range [1–5].

NES	s_ij_	Condition
1	$s_{ij}=\max(s_{i})$	$\min(s_{i})\neq \max(s_{i})$
0.50	s_ij_ = 4	$\min(s_{i})=1$, $\max(s_{i})=5$
0.33	*s*_*ij*_ = 3	$\min(s_{i})=1$, $\max(s_{i})=4$
	*s*_*ij*_ = 4	$\min(s_{i})=2$, $\max(s_{i})=5$
0	$s_{ij}=1,\ldots ,5$	$\min(s_{i})=\max(s_{i})$
	$s_{ij}=\left[\frac{\min(s_{i})+\max(s_{i})}{2}\right]$	for each $\min(s_{i})$ and $\max(s_{i})$
−0.33	*s*_*ij*_ = 2	$\min(s_{i})=1$, $\max(s_{i})=4$
	*s*_*ij*_ = 3	$\min(s_{i})=2$, $\max(s_{i})=5$
-0.50	*s*_*ij*_ = 2	$\min(s_{i})=1$, $\max(s_{i})=5$
-1	$s_{ij}=\min(s_{i})$	$\min(s_{i})\neq \max(s_{i})$

It is important to note that NES is designed to be robust to skewed distributions. Even if the observed rating range for an item is narrow (e.g., ratings vary only between 4 and 5), a rating falling on the boundary of this range will still yield a maximal NES value (±1). For instance, if an item’s ratings range from 4 to 5, the midpoint is 4.5; a rating of 5 results in a NES of +1 (i.e., (5−4.5)/0.5). Thus, the index correctly identifies the rating as extreme relative to the available context, preventing the underestimation of extremity in polarized product distributions.

Generally:

- *NES* = 0 denotes a completely non-extreme review and occurs either when the review matches the midpoint of the observed rating range, or when all ratings for the item are equal and consequently the minimum and maximum values coincide (i.e., $\min(s_{i})=\max(s_{i})$).
- *NES* = +1 indicates an extreme positive review, meaning that the reviewer assigned the highest rating observed for the item. Conversely, NES=−1 corresponds to an extreme negative review, where the reviewer assigned the lowest rating. This interpretation holds unless all ratings for the item are identical, in which case the *NES* is equal to 0, reflecting the absence of any extremity.
- Intermediate values such as ±0.50 or ±0.33 emerge from specific combinations of rating and range and establish distinct degrees of deviation from the midpoint of the observed rating range for the item. For example, a *NES* of 0.50 corresponds to a rating that lies 50% above the midpoint of the observed rating range for the item, indicating a moderate level of positive extremity.

As shown in Table 2, the same rating *s*_*ij*_ may be perceived as extreme or not depending on the range of ratings the item has received from all reviewers. Consequently, the *NES* does not convey the same information as the raw rating; instead, it functions as a metric that captures the extent to which a review deviates from the midpoint of the item’s observed rating range.

### 3.2 The reviewer extremeness index (REI)

To move beyond the analysis of individual reviews and capture a reviewer’s overall rating behavior, we introduce the Reviewer Extremeness Index (REI). This metric quantifies the proportion of extreme ratings (either positive or negative) relative to the total number of reviews provided by reviewer *j*, defined as reviews with a *NES* equal to either +1 or −1.

Let *n*_*j*_ denote the total number of reviews submitted by reviewer *j*. The REI for reviewer *j* is defined as:


$$REI_{j}=\frac{1}{n_{j}}\sum_{i=1}^{n_{j}}𝕀(|NES_{ij}|=1)$$ (2)

where $𝕀(|NES_{ij}|=1)$ is an indicator function that returns 1 if the *i*-th review by reviewer *j* is extreme (i.e., |*NES*_*ij*_| = 1), and 0 otherwise.

The REI thus takes values in the interval [0,1], where 1 indicates that all of the reviewer’s ratings are extreme, and 0 means none are.

In this way, the REI provides a normalized and interpretable measure of a reviewer’s tendency toward extreme evaluations across their review history.

### 3.3 The reviewer polarity index (RPI)

To capture the directional tendency of a reviewer’s extreme ratings, we introduce the Reviewer Polarity Index (RPI). This metric adapts a well-established formula for polarity scoring, commonly used in sentiment analysis and computational linguistics [33–35], to the specific context of rating extremity. The RPI is calculated as the normalized difference between the count of positive and negative extreme ratings. Unlike binary comparisons or ratios—which may lead to undefined values when denominators approach zero—we compute the normalized difference between the number of positive and negative extreme ratings, relative to their total.

Let $n_{j}^{+}$ denote the number of extreme positive ratings (*NES*_*ij*_ = +1) and $n_{j}^{-}$ the number of extreme negative ratings ($NES_{ij}=-1$) provided by reviewer *j*. The RPI is formally defined as:


$$\begin{array}{c} RPI_{j}=\left\{ \begin{array}{cc} \frac{n_{j}^{+}-n_{j}^{-}}{n_{j}^{+}+n_{j}^{-}} & \text{if }n_{j}^{+}+n_{j}^{-}>0\\ \text{undefined} & \text{if }n_{j}^{+}+n_{j}^{-}=0 \end{array}\right.\; \end{array}$$ (3)

The *RPI* operates on a continuous scale from −1 to +1, providing a nuanced measure of a reviewer’s tendency toward extreme positivity or negativity. An RPI of +1 indicates that all extreme ratings provided by a reviewer are positive ($n_{j}^{-}=0$), while an RPI of −1 reflects the opposite case—all extreme ratings are negative ($n_{j}^{+}=0$). An RPI of 0 denotes a perfect balance between extreme positive and extreme negative ratings ($n_{j}^{+}=n_{j}^{-}$), suggesting a neutral polarity.

Values between these extremes convey the degree and direction of imbalance: positive RPI values signal a predominance of extreme positive ratings over negative ones, and negative values indicate the reverse.

Consider the following examples:

- If a reviewer has $n_{j}^{+}=5$ and $n_{j}^{-}=5$, then:

$$RPI_{j}=\frac{5-5}{5+5}=\frac{0}{10}=0$$

indicating a neutral polarity.

- If a reviewer has $n_{j}^{+}=6$ and $n_{j}^{-}=4$, then:

$$RPI_{j}=\frac{6-4}{6+4}=\frac{2}{10}=+0.20$$

indicating a slightly positive polarity.

- If a reviewer has $n_{j}^{+}=9$ and $n_{j}^{-}=1$, then:

$$RPI_{j}=\frac{9-1}{9+1}=\frac{8}{10}=+0.80$$

indicating a strongly positive polarity.

### 3.4 Modeling reviewer behavior in two dimensions: REI and RPI

The two indices, REI and RPI, capture fundamental and complementary aspects of reviewer behavior:

- REI measures the frequency with which a reviewer assigns extreme ratings (with NES=±1). It answers the question: *“What proportion of this user’s reviews are extreme?”*
- RPI measures the direction and intensity of the imbalance between positive and negative extremes. It addresses the question: *“When this reviewer gives extreme ratings, in which direction do they lean and how strongly?”*

**Fig 1:**
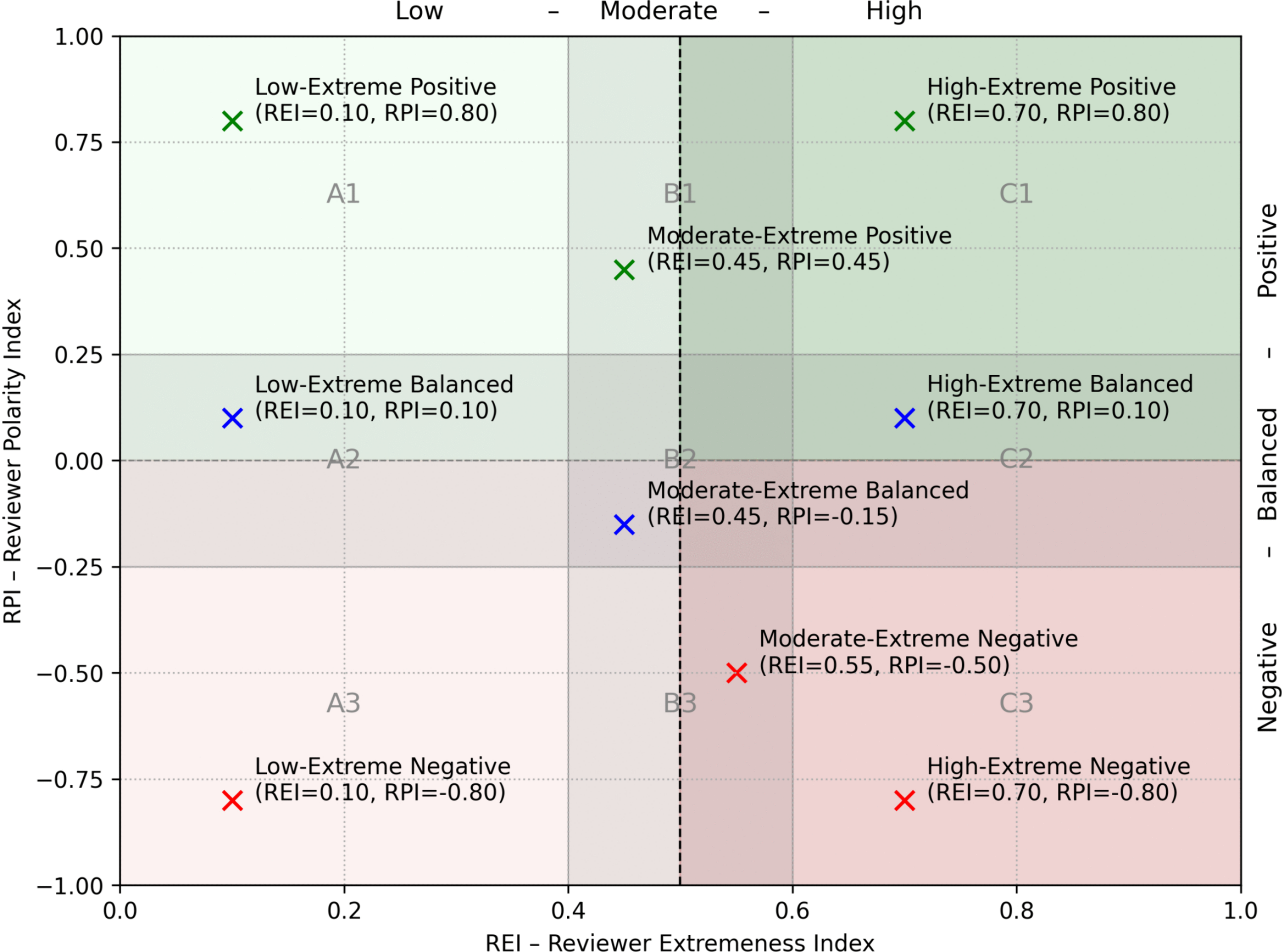
Reviewer behavioral profiles.

The combination of these two indices allows each reviewer to be mapped onto a two-dimensional space, where REI and RPI serve as orthogonal axes. This structured and nuanced representation enables the systematic identification of distinct reviewer profiles. As shown in Fig 1, the two-dimensional space is divided by two shaded central bands:

- A *vertical* band, for REI values between 0.4 and 0.6, delineates the zone of reviewers with a ‘moderate frequency of extreme ratings’. These thresholds were selected to conceptually distinguish between those who use extreme ratings only occasionally (*REI* < 0.4) and those for whom such ratings represent a systematic and distinctive feature of their reviewing style (*REI* > 0.6).
- A *horizontal* band, for RPI values between −0.25 and +0.25, is defined as the zone of ‘substantial balance’. It groups reviewers with neutral polarity, whose extreme ratings are directionally balanced between positive and negative. A reviewer falling within this range does not exhibit strong polarity, maintaining a relatively balanced ratio between positive and negative extreme reviews (for example, an RPI of 0.25 corresponds to five positive reviews for every three negative ones).

These bands partition the plane into nine subregions, which can be grouped into three main families based on polarity (RPI).

#### Positive polarity profiles (*RPI* > 0.25).

These reviewers tend to express extreme ratings in a positive direction. Their enthusiasm varies depending on how frequently they display it.

- *A1: Low-Extreme Positive* – Reviewers who rarely use extreme ratings, but when they do, they are predominantly positive. Due to their selectivity, their rare highly positive reviews may carry particular significance.
- *B1: Moderate-Extreme Positive* – Reviewers with a moderate frequency of extreme ratings and a positive polarity. This profile may reflect a generally favorable attitude tempered by selective enthusiasm.
- *C1: High-Extreme Positive* – Users who frequently assign extreme ratings, with a strong bias toward positivity. This group may include highly enthusiastic individuals or, potentially, users involved in incentivized reviewing practices. This group therefore represents a segment of users whose behavior warrants closer examination, as will be discussed in detail in Sect 6.

#### Balanced polarity profiles (−0.25≤ RPI ≤+0.25).

These reviewers show no directional bias, using extreme ratings both positively and negatively, in approximately equal measure. They are often perceived as reliable sources due to their apparent impartiality.

- *A2: Low-Extreme Balanced* – Reviewers who seldom assign extreme ratings and show no clear preference between positive and negative. Their behavior suggests caution and neutrality.
- *B2: Moderate-Extreme Balanced* – Reviewers who use extreme ratings with moderate frequency in both directions. A moderate REI combined with a near-zero RPI indicates critical consistency and discernment, reflecting the ability to “call things as they are.” These users are often among the most informative and trustworthy contributors.
- *C2: High-Extreme Balanced* – Reviewers in this category frequently assign extreme ratings in both directions, making them appear assertive and informative. However, this persistent use of polar opposite ratings reduces the nuance of their feedback. This stands in contrast to B2 reviewers, who provide more granular feedback by employing a wider range of rating scores.

#### Negative polarity profiles (RPI<−0.25).

These reviewers tend to express ratings in a negative direction. Their critical stance is a defining characteristic.

- *A3: Low-Extreme Negative* – Users who rarely post extreme ratings, but when they do, they are predominantly negative. Their rare positive feedback may thus be seen as especially credible and noteworthy.
- *B3: Moderate-Extreme Negative* – Reviewers with a moderate use of extreme ratings, clearly skewed toward negative evaluations. They may offer a critical perspective without being excessively harsh.
- *C3: High-Extreme Negative* – Users who frequently assign extreme ratings with a strong bias toward negativity. This reviewing style may reflect high expectations, strong critical rigor, or deliberately punitive behavior.

This nine-profile taxonomy provides a unique and interpretable ‘behavioral signature’ for every reviewer, derived entirely from their rating history. Ultimately, the framework digitizes the fundamental human heuristic of ‘knowing the source’, providing a clear behavioral context through which to interpret any single review.

### 3.5 Validation and robustness checks

Although the REI and RPI indices are formally defined and fully deterministic, it remains essential to assess their validity and reliability as behavioral measures. To this end, we implemented a set of complementary strategies on the case study data:

- *Convergent and discriminant validity*: We compared the REI and RPI values with a relative indicator of extremity already discussed in the literature, namely the variance of reviewer ratings, which measures how much a reviewer ‘oscillates’ in the scores assigned to different items relative to their own mean. Since a reviewer who consistently assigns extreme ratings (e.g., always 5 stars) exhibits a high REI but zero variance, we analyzed the relationship between these metrics to confirm that the proposed framework captures behavioral patterns that are distinct from simple dispersion (discriminant validity).
- *Stability and test–retest reliability*: For a subset of reviewers with long activity spans, we computed REI and RPI separately over non-overlapping temporal windows. The indices showed consistent values across different time periods, indicating that they capture stable reviewing tendencies rather than short-term fluctuations.
- *Robustness to subsampling*: We applied bootstrapping procedures by randomly resampling reviewer histories. The resulting distributions of REI and RPI remained highly consistent, demonstrating the robustness of the framework against data perturbations.

These analyses, reported in the case study, strengthen the construct validity of the REI–RPI framework, showing that it is both interpretable and empirically stable, while at the same time providing unique explanatory power compared to existing one-dimensional measures of rating extremity.

## 4 Case study

### 4.1 The input data

The dataset used in this study was obtained from the public Kaggle repository (https://www.kaggle.com/datasets/mohamedbakhet/amazon-books-reviews/data) [36]. Data collection and analysis comply with the terms and conditions of the source platform. The dataset consists of approximately 3 million book reviews written by over 1 million users for 212,404 different titles. This dataset is part of the Amazon Review corpus, which includes review content and metadata from May 1996 to July 2014. Table 3 reports the key variables extracted and basic descriptive statistics.

**Table 3:** Features description of the Amazon Books Dataset.

Features	Description	Unique	Null	Total
		values	values	values
Id	The Id of Book	221,998	0	3,000,000
Title	Book Title	212,403	208	2,999,792
Price	The price of Book	6,004	2,518,829	481,171
User_id	Id of the user who rates the book	1,008,972	561,787	2,438,113
profileName	Name of the user who rates the book	854,146	561,886	2,438,114
review/helpfulness	Helpfulness rating of the review, e.g., 2/3	12,084	0	3,000,000
review/score	Rating from 1 to 5 for the book	5	0	3,000,000
review/time	Time when the review was given	6,272	0	3,000,000
review/summary	The summary of a text review	1,592,315	38	2,999,962
review/text	The full text of a review	2,062,648	8	2,999,992

To ensure meaningful analysis of reviewer-level behavior, we performed several preprocessing steps. We removed reviews that lacked either a book title or a user ID, as these were essential for identifying unique reviewer-item pairs. We also eliminated duplicate reviews from the same reviewer on the same book, keeping only one instance. To ensure the stability of the extremeness and polarity measures, we excluded reviewers who had authored fewer than three reviews. Consistent with the long-tail distribution typical of online activity, this threshold reduced the initial pool of 1,008,972 unique user identifiers to an analytic sample of 143,107 active reviewers (representing approximately 14.2% of the total user base). This subset, however, accounts for the majority of the platform’s sustained social interaction and content generation.

### 4.2 Results

**Fig 2:**
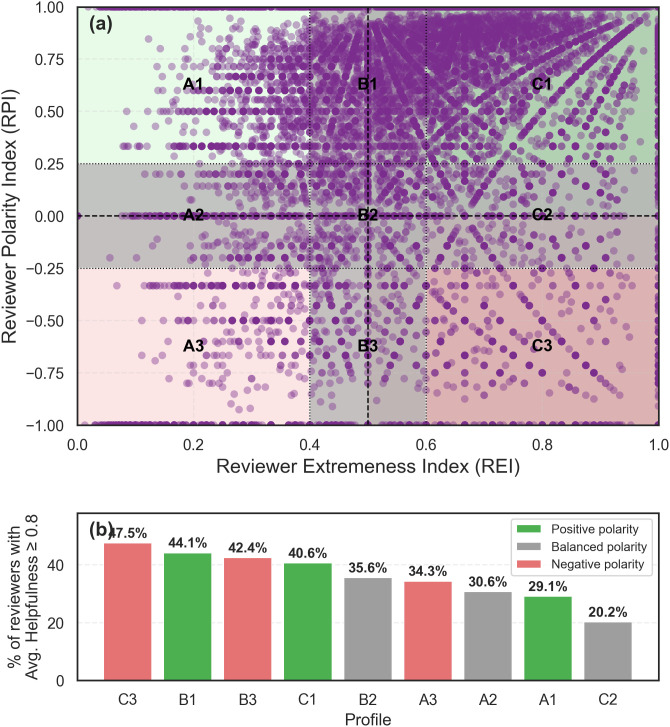
Scatterplot of reviewer behavior based on REI and RPI metrics. **(a)** Scatterplot of all reviewers in the REI–RPI space, showing the concentration of the reviewer population across the nine profile zones. **(b)** Proportion of highly helpful reviewers (Avg. Helpfulness Ratio ≥ 0.8) within each profile zone (corresponding to the rightmost column of Table 4). The two panels are complementary: panel (a) shows that C1 dominates in absolute numbers, while panel (b) reveals that C3 exhibits the highest proportion of helpful reviewers (47.51%), followed by B1 (44.14%).

Fig 2 maps the distribution of individual reviewers within the REI-RPI space, while Table 4 provides a detailed quantitative breakdown. The combined analysis of both reveals remarkably clear distributional and behavioral patterns.

**Table 4:** Distribution of reviewers across profiles, sorted by total reviewer percentage.

Profile	Total Reviewers		Reviewers with avg. Helpfulness ≥0.8	
	Count	%	Count	% of zone
C1	72,691	50.79	29,525	40.62
A1	20,505	14.33	5,961	29.07
B1	13,805	9.65	6,093	44.14
C3	10,599	7.41	5,036	47.51
C2	10,111	7.07	2,038	20.16
A3	5,426	3.79	1,862	34.32
B2	5,267	3.68	1,873	35.56
B3	2,762	1.93	1,172	42.43
A2	1,941	1.36	595	30.65
Total	143,107	100	54,155	–

An examination of the overall distribution (Table 4) shows a marked tendency toward positive polarization. The A1 (Low-Extreme Positive), B1 (Moderate-Extreme Positive), and C1 (High-Extreme Positive) profiles together account for 75% of the total reviewer population. The C1 category is particularly dominant, alone comprising more than half (50.79%) of all reviewers. Conversely, profiles with negative polarity (A3, B3, and C3) are considerably less populated, collectively representing only 13.13% of the sample, which confirms a general inclination toward positivity in ratings. While Fig 2(a) illustrates this distributional pattern, Fig 2(b) complements it by showing the proportion of highly helpful reviewers within each zone, revealing a markedly different ranking.

Integrating these findings with data on perceived utility (the helpfulness ratio) offers deeper insights. Table 4 shows that reviewers with high helpfulness scores (≥0.8) are present across all profile zones, though unevenly distributed. In absolute terms, the C1 group contains the largest number of highly helpful reviewers (29,525 out of 54,155), which is expected given that this zone alone comprises over 50% of the reviewer population.

One of the most notable findings concerns profile C3 (High-Extreme Negative). As shown in Fig 2(b), this zone exhibits the highest proportion of highly helpful reviewers (47.51%), followed by B1 (44.14%) and B3 (42.43%). This result is particularly significant, as it shows that reviewers who adopt a consistently critical stance—likely perceived as rigorous auditors acting as a ‘warning system’ for potential buyers—are highly valued by the community, often more so than consistently positive reviewers.

An important aspect to consider is how the perceived helpfulness of a review may change when additional information about the reviewer’s behavioral tendencies is made explicitly available. If platforms were to disclose a reviewer’s profile—such as identifying them as an Extreme Positive Reviewer—this contextual metadata could influence how readers interpret the content and credibility of the review. A uniformly enthusiastic rating, for example, may be assessed differently if it is known to come from a reviewer who consistently gives highly positive evaluations. This highlights the unique contribution of the REI–RPI framework: by making underlying reviewer biases visible, it enables platforms to provide contextual signals that can inform how reviews are interpreted, prioritized, or weighted in ranking algorithms.

### 4.3 Simulation of framework implementation

To assess the practical implementability of the REI-RPI framework within a real-world e-commerce environment, we addressed the challenge of conveying complex behavioral data without overburdening the end user. While the nine archetypal profiles provide essential granularity for internal platform monitoring and recommendation algorithms, displaying raw indices or a complex grid to consumers could lead to information overload.

Therefore, we propose a dual-layer implementation strategy:

- Back-end (Platform level): The system retains the full nine-profile granularity to refine ranking algorithms and detect anomalous patterns (e.g., incentivized reviews).
- Front-end (User level): The information is synthesized into intuitive visual cues that require no specific user training.

**Fig 3:**
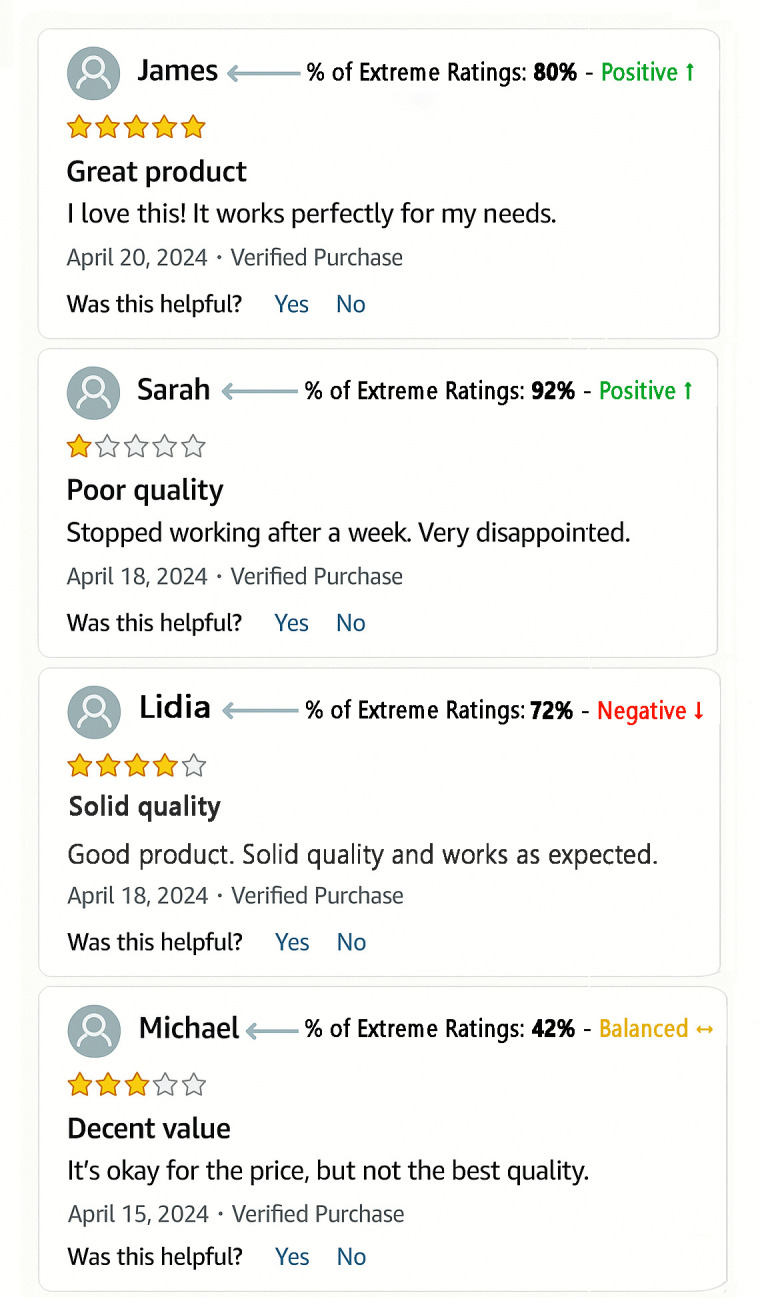
Simulated implementation of the REI-RPI framework on a review platform. The REI is presented as the ‘% of Extreme Ratings’, whereas the RPI is expressed through a polarity label and a directional symbol (Positive ↑ , Balanced ↔ , Negative ↓). As illustrated, this behavioral badge provides immediate context to the reader: Sarah’s rare 1-star review stands out against her usually positive profile (92% ↑), while Lidia’s generally critical profile (72% ↓) lends strong credibility to her positive assessment. *Mock-up created by the authors for illustrative purposes*.

Fig 3 presents a mock-up simulation of this interface, demonstrating how the REI and RPI can be integrated as user-facing metrics. Each reviewer is accompanied by a numerical label (‘% of Extreme Ratings’) conveying the REI, and a Polarity Badge conveying the RPI. These cues enable consumers to contextualize individual ratings at a glance. For instance, Sarah’s 1-star review acquires a profoundly different meaning when the reader learns that 92% of her ratings are extreme and positively oriented: rather than being dismissed as the complaint of a chronically dissatisfied user, this rare negative assessment likely reflects a genuinely unsatisfactory experience. Conversely, Lidia’s 4-star rating carries added weight; given her predominantly critical history (72% extreme, Negative ↓), a positive score from her acts as a strong signal of quality. In both cases, the behavioral badge transforms an isolated rating into an easily interpretable signal.

## 5 Validation and robustness results

### 5.1 Internal validation

To assess the empirical validity of the REI–RPI framework, we implemented complementary checks on the case study data.

First, convergent and discriminant validity analyses compared REI and RPI with the reviewer-level variance of ratings. As expected, REI showed only a modest association with variance (ρ=−0.27,p<0.001), while RPI showed a similarly low association with variance (ρ=−0.33,p<0.001), confirming that the two indices capture distinct behavioral dimensions rather than generic variability.

Second, robustness to subsampling was tested by randomly removing 20% of reviews from each reviewer’s history and recomputing REI and RPI (three replications). The results remained highly stable, with mean absolute deviations around 0.060 for REI and 0.065 for RPI, and median deviations close to zero, demonstrating that the framework is resistant to random perturbations of the data.

Third, we assessed temporal stability by splitting the reviewing history of users with at least 10 reviews and a span of more than two years into two non-overlapping windows. For 15,770 reviewers meeting this criterion, REI showed moderate consistency across time (ρ=0.31, mean absolute deviation = 0.24), while RPI also displayed positive correlation across periods (ρ=0.37, mean absolute deviation = 0.41). Although individual values may fluctuate, the results indicate that both indices preserve significant stability over time, further supporting the construct validity of the framework.

Finally, to further assess the generalizability of the proposed framework beyond the book domain, we replicated the profiling analysis using the MovieLens dataset (movies category). The results, provided in Supplementary Information S2 (S2 Appendix), demonstrate that the REI and RPI indices remain robust and effectively identify reviewer archetypes even in a different rating ecosystem with distinct distribution characteristics.

### 5.2 External validation

To establish the external validity of the framework, we tested whether the proposed indices (REI and RPI) predict meaningful outcomes in the ecosystem, specifically the perceived utility of reviews (Helpfulness). We conducted an Ordinary Least Squares (OLS) regression on 118,270 reviewers (reviewers with at least one helpfulness vote). Although the dependent variable (Mean Helpfulness Ratio) is bounded between 0 and 1, which might theoretically warrant a Fractional Logistic/Probit model, we opted for an Ordinary Least Squares (OLS) approach. Given the large sample size (*N* > 118,000), OLS provides reliable estimates and, crucially, offers superior interpretability of the coefficients as direct marginal effects. This approach aligns with standard methodological practices in large-scale empirical studies where the primary goal is to assess the direction and significance of relationships rather than boundary predictions.

The dependent variable was the reviewer’s Mean Helpfulness Ratio. The independent variables were REI and the absolute value of RPI (to measure polarization magnitude regardless of direction). We controlled for review length (log_length) and reviewer activity (log_activity). For the regression analysis, we restricted the sample to reviewers who had received at least one helpfulness vote and for whom the RPI was defined, i.e., those with at least one extreme rating (NES=±1). Reviewers with no extreme ratings ($n_{j}^{+}+n_{j}^{-}=0$) were therefore excluded from this analysis.

As shown in Table 5, the analysis reveals that both indices are statistically significant predictors of helpfulness (*p* < 0.001). Specifically, the positive coefficient for REI (β=0.025) suggests that reviewers who adopt extreme rating styles are perceived as more informative. Notably, the polarization intensity (RPI) shows a strong positive effect (β=0.066), indicating that the community perceives consistent reviewers (whether strictly critical or enthusiastic) as significantly more helpful than ambivalent ones. This confirms that the behavioral profiles mapped by the framework capture traits that are economically and socially relevant within the platform.

**Table 5:** OLS regression results. Dependent Variable: Mean Helpfulness Ratio (*N* = 118,270, *R*^2^ = 0.085).

Variable	coef. *β*	Std. Error	t	*P* > |*t*|
Intercept	0.1345	0.006	23.792	0.000
REI	0.0254	0.003	9.163	0.000
RPI_abs	0.0661	0.002	27.987	0.000
log_activity	0.0014	0.001	1.028	0.304
log_length	0.1034	0.001	98.376	0.000

Interestingly, the volume of reviewer activity (log_activity) was not a significant predictor (*p* = 0.304). This suggests that a reviewer’s accumulation of experience (quantity) does not inherently translate into perceived utility. Instead, the effort put into individual reviews (proxied by log_length, β=0.103,p<0.001) and the adoption of a clear behavioral stance (high REI and RPI) are the primary drivers of community recognition. This highlights the unique contribution of the REI-RPI framework in capturing qualitative behavioral traits that traditional volume metrics overlook.

In this analysis, while the reported beta coefficients for REI (β=0.025) and RPI (β=0.066) may initially appear modest, they indicate a tangible impact on perceived utility. Specifically, a complete shift from a balanced to a fully polarized profile (RPI moving from 0 to 1) is associated with a 6.6 percentage point increase in the helpfulness ratio. Given the baseline helpfulness intercept of approximately 13.5%, this behavioral consistency effectively boosts a reviewer’s expected utility by nearly 50% relative to the baseline (0.066/0.135), underscoring the community’s preference for decisive viewpoints. Similarly, transitioning from a reviewer who never uses extreme ratings to one who uses them exclusively yields a 2.5 percentage point increase in helpfulness. Relative to the baseline, this represents an approximate 18% relative increase in perceived utility (0.025/0.135).

Finally, to address the bounded nature of the dependent variable ([0,1]), we validated our findings using a Fractional Logistic Regression model (GLM specified with a quasibinomial family and logit link). This complementary analysis yielded coefficients with the same direction and statistical significance as the primary OLS model, fully confirming the robustness of the reported estimates.

### 5.3 Cross-domain generalizability

To further assess the robustness of the proposed framework beyond the e-commerce book domain, we addressed the issue of generalizability across different platforms. As noted by Schoenmueller et al. [4], rating distributions can vary significantly depending on the context (e.g., retail vs. entertainment). To test the framework’s adaptability, we replicated the profiling analysis using the MovieLens 100K dataset, a widely used benchmark for movie ratings.

The results of this supplementary analysis are detailed in Supplementary Information S2 (S2 Appendix). The application of the REI–RPI indices to this new domain revealed a distinct behavioral landscape compared to Amazon, confirming the framework’s sensitivity to platform-specific dynamics while retaining its ability to profile and segment users effectively. This validates that the mathematical properties of the indices are platform-agnostic and capable of adapting to diverse rating ecosystems.

## 6 Discussion

In this paper, we introduced a novel and intuitive two-dimensional framework for profiling reviewer behavior through two complementary indices: the Reviewer Extremeness Index (REI), which quantifies the frequency of extreme ratings, and the Reviewer Polarity Index (RPI), which measures their directional balance and intensity. By mapping reviewers onto the REI–RPI plane, our approach generates a distinctive *behavioral signature* that offers significant benefits for both consumers and online retail platforms, enhancing transparency and reliability within the digital marketplace ecosystem.

### Implications for consumers

The primary value of the REI–RPI framework for consumers lies in its ability to provide crucial contextual information that goes beyond the face value of individual ratings. Knowing a reviewer’s archetype allows for a more informed and nuanced interpretation of their feedback. For instance, a glowing review from a user identified as a *Low-Extreme Negative* reviewer (low frequency of extreme rating and negative polarity) is likely to carry greater informational weight and credibility than an identical review from an *Extreme Positive* reviewer (high frequency of extreme rating and positive polarity).

This addresses a key flaw in many rating systems, where all reviews are presented as equally informative regardless of the reviewer’s history or tendencies. As demonstrated in our simulation (Sect 4.3 and Fig 3), this enhanced transparency can be effectively operationalized through simple visual cues—such as ‘Polarity Badges’ or extremeness percentages—integrated directly into the user interface. By making these behavioral signals visible at a glance, the framework empowers consumers to adjust their trust levels dynamically without requiring complex analysis.

### Implications for online retail platforms

For platforms, the REI–RPI framework offers a simple, scalable, and interpretable tool for monitoring user behavior. One of its core strengths is that it relies solely on numerical rating data, making it computationally efficient and easy to implement at scale across millions of users—without requiring complex textual analysis.

From a practical standpoint, identifying reviewer profiles enables platforms to optimize their content strategy. As demonstrated by recent studies, segmenting reviewers based on their behavioral history can significantly enhance the prediction of market demand [37]. To operationalize this, we analyzed three key business metrics across the identified profiles: productivity (average reviews per reviewer), perceived quality (average helpfulness ratio), and information depth (average review length). The results, summarized in Table 6, reveal a crucial functional dichotomy in the reviewer community:

- *The ’Engaged Analysts’ (Zone B1-B2):* Contrary to the assumption that extreme reviewers drive platform volume, our analysis shows that *reviewers who employ extreme ratings with moderate frequency* (Zone B, or ‘Moderate-Extreme’) are the most productive contributors. Specifically, B1 reviewers contribute an average of 18.88 reviews per reviewer, and B2 reviewers write among the longest and most detailed texts (188 words on average). These reviewers represent the ‘engaged core’ of the platform, combining high productivity with high community appreciation (helpfulness ratios ≈ 0.75).
- *The ’Quick Signalers’ (Zone C1):* While *reviewers who habitually assign extreme ratings* (Zone C1, ‘High-Extreme’ Positive), constitute the largest demographic (51% of reviewers; see Table 4), they are individually less productive (7.1 reviews/reviewer) and provide the shortest feedback (126 words). Their contribution lies primarily in providing quick, low-friction sentiment signals rather than deep analytical content.

**Table 6:** Business Metrics by Reviewer Profile (Sorted by Quality).

Profile	Volume	Quality	Detail
	(Avg. Reviews per reviewer)	(Avg. Helpfulness ratio)	(Avg. Review length)
B1	18.88	0.75	181.01
B2	13.54	0.73	188.12
C1	7.10	0.73	126.54
A1	8.32	0.72	155.32
C2	6.17	0.68	162.09
A3	7.06	0.67	171.89
B3	9.18	0.66	188.24
A2	5.36	0.60	143.62
C3	4.60	0.52	140.64

This distinction allows platforms to refine recommendation algorithms: reviews from **B-Zone** profiles should be prioritized when consumers seek detailed decision-support information, whereas **C-Zone** ratings are valuable for generating aggregate sentiment scores.

Moreover, regarding anomaly detection, our framework aligns with findings that non-textual behavioral patterns often offer superior robustness compared to purely text-based models [38]. By leveraging the REI-RPI coordinates, platforms can implement scalable pre-screening filters to identify systematic outliers—such as users with maximum REI and fixed Polarity who deviate from the productivity norms of their cluster—before deploying computationally expensive network analyses. Platforms can leverage this framework to:

- *Enhance transparency:* Displaying reviewer archetypes (e.g., through badges or on profile pages) can empower consumers to make better-informed decisions directly on the platform.
- *Identify problematic behavior:* The framework can effectively flag profiles that may warrant further scrutiny. It is essential to emphasize that the REI–RPI framework is a tool for signaling behavioral patterns, not a definitive instrument for detecting misconduct. A profile such as ’High-Extreme Positive’ (C1), particularly when associated with unusually high helpfulness scores but low review detail, does not in itself constitute evidence of incentivized reviewing. Rather, it serves as a warning signal that calls for closer examination. The value of the framework lies in its ability to function as an efficient and scalable preliminary filter.

It is crucial to distinguish our framework from metrics such as Amazon’s *helpfulness* score. While the helpfulness ratio reflects the perceived utility of a single review based on peer feedback, the REI–RPI framework captures the reviewer’s historical behavior. The two are complementary: our approach provides insight into the credibility of the source—much like in real-world settings, where advice is evaluated in light of the speaker’s known tendencies.

### Limitations and conclusions

A primary limitation of the REI-RPI framework is its dependence on a sufficient history of past reviews to generate a reliable profile. Online review ecosystems structurally exhibit a long-tail distribution of user activity, where a small core of ’power users’ contributes a disproportionate volume of content, while the vast majority of consumers post reviews only sporadically. As transparently reported in our data description, applying the minimum threshold of three reviews required to compute ratio-based metrics resulted in the exclusion of approximately 86% of the unique user identifiers in our dataset.

Consequently, the framework is subject to the ’cold-start’ problem: it cannot generate a behavioral signature for new users or ’one-time’ reviewers until they accumulate a minimum engagement history. For these users, the platform would need to rely on traditional item-level metrics (e.g., helpfulness votes on the single review) rather than reviewer-level profiling. It is important to note, however, that this constraint is not unique to the REI and RPI indices; rather, it is an inherent limitation of any metric aiming to assess behavioral consistency, trustworthiness, or longitudinal reputation. Future implementations could address this by integrating hybrid models that default to text-based sentiment analysis for cold-start users and transition to REI-RPI profiling as the user’s history grows.

A central limitation of our framework is that it focuses exclusively on numerical ratings, without considering the accompanying textual content of reviews. While this may appear restrictive, there are several reasons why numerical-only analysis represents a valid and useful approach in its own right. First, star ratings are the only element systematically available across virtually all platforms and for every review, ensuring scalability and comparability [1]. Second, numerical ratings are directly integrated into recommendation algorithms, average scores, and ranking systems, which makes them the most consequential input for both consumers and platforms [25]. Third, a focus on rating extremity allows us to abstract from the semantic richness of the text and isolate structural behavioral tendencies that persist regardless of content. Moreover, prior research has consistently shown a high degree of concordance between star ratings and textual sentiment [5,35], supporting the validity of a rating-based approach.

We acknowledge that textual data can offer valuable complementary insights—for instance, to distinguish between well-argued criticism and opportunistic negativity. However, our framework is deliberately designed to remain computationally lightweight, interpretable, and platform-agnostic, attributes that would be diluted if complex NLP pipelines were required. Future research can extend the REI–RPI approach by integrating textual signals, but its contribution as a standalone rating-based tool lies precisely in its ability to scale and generalize across datasets and contexts.

Finally, future research should assess the practical implementation of these indicators from a user experience (UX) perspective. Conducting A/B testing on live platforms would be a valuable next step to measure how different visual representations of the REI-RPI archetypes influence consumer trust and decision-making in real-time.

In a digital landscape increasingly shaped by user-generated content, understanding the structure of reviewer behavior is essential. The REI–RPI framework represents a meaningful step in this direction. By moving beyond aggregated single metrics and offering a transparent, multidimensional view of reviewer tendencies, it empowers consumers, equips platforms with a valuable monitoring tool, and ultimately contributes to a more reliable and trustworthy online marketplace.

## Supporting information

S1 Appendix Proof of NES. Demonstration that *NES* is a normalized score ranging from −1–1.
(PDF)

S2 Appendix Framework Generalizability. Application to the MovieLens Dataset
(PDF)

S3 File Analysis Code. Jupyter notebook for REI-RPI framework analysis and to reproduce the results.
(ZIP)
