# Supplementary material for: A two-dimensional framework for profiling online reviewer behavior
Source: PLoS One. 2026 Mar 25;21(3):e0344988. doi: 10.1371/journal.pone.0344988 (PMC13016354; doi:10.1371/journal.pone.0344988)
Supplement: S1 Appendix — Demonstration that NES is a normalized score ranging from −1–1. (PDF) [file pone.0344988.s001.pdf]

S1 Proof of NES

We need to demonstrate that NES is a normalized score ranging from -1 to $+1$. Consequently, the maximum value the numerator of the NES formula can assume is equal to the denominator.

The NES formula is:

$$NES_{ij} = \frac{s_{ij} - \frac{min(s_i)+max(s_i)}{2}}{\frac{max(s_i)-min(s_i))}{2}}$$

For simplicity, let's denote $min(s_i)$ as a and $max(s_i)$ as b. The formula becomes:

$$NES_{ij} = \frac{s_{ij} - \frac{a+b}{2}}{\frac{b-a}{2}} = \frac{2s_{ij} - (a+b)}{b-a}$$

The function representing the numerator is:

$$f(s_{ij}) = 2s_{ij} - (a+b)$$

We need to find the maximum value of this function $f(s_{ij})$. So, we compute the first derivative:

$$f'(s_{ij}) = \frac{d}{ds_{ij}}\left(2s_{ij} - (a+b)\right) = 2$$

Since the derivative $f'(s_{ij})$ is a positive constant, the function $f(s_{ij})$ is strictly increasing. To find the maximum value, we evaluate $f(s_{ij})$ at the boundaries of the interval $[a, b]$:

- When $s_{ij} = a$

$$f(a) = 2a - (a+b) = a - b$$

  Its absolute value is :$|a - b| = b - a$

- When $s_{ij} = b$

$$f(b) = 2b - (a+b) = b - a$$

Particularly, from the previous step: $|2s_{ij} - (a+b)| = b - a$, that is exactly equal to the denominator of NES simplified formula: $b - a$.

- at $s_{ij} = a$ the

$$NES = \frac{a-b}{b-a} = -1$$

- at $s_{ij} = b$ the

$$NES = \frac{b-a}{b-a} = 1$$

In conclusion, we have thus demonstrated that the numerator can indeed reach the maximum value of the denominator, proving that the index is normalized and varies between -1 and 1.
