# Supplementary material for: A two-dimensional framework for profiling online reviewer behavior
Source: PLoS One. 2026 Mar 25;21(3):e0344988. doi: 10.1371/journal.pone.0344988 (PMC13016354; doi:10.1371/journal.pone.0344988)
Supplement: S2 Appendix — Application to the MovieLens Dataset (PDF) [file pone.0344988.s002.pdf]

S2 Framework Generalizability: Application to the MovieLens Dataset

To assess the robustness and generalizability of the REI-RPI framework beyond the book review domain, we replicated the profiling analysis using the MovieLens 100K dataset, a widely used benchmark in the recommendation systems literature focusing on movie ratings.

Data Description The dataset consists of 100,000 ratings (on a scale of 1 to 5) from 943 users on 1,682 movies. The data was collected through the MovieLens website (`grouplens.org`) over a seven-month period. Unlike the Amazon Books dataset, which is derived from a transactional e-commerce environment, MovieLens represents a dedicated entertainment recommendation platform, characterized by different user motivations and rating behaviors.

Dataset Source: `https://grouplens.org/datasets/movielens/100k/`

Dataset reference citation: Harper, F. M., & Konstan, J. A. (2015). The MovieLens Datasets: History and Context. ACM Transactions on Interactive Intelligent Systems (TiiS), 5(4), 1—19. DOI: `https://doi.org/10.1145/2827872`.

A Results and Discussions

We applied the identical processing pipeline described in the main manuscript:

- **NES Calculation:** The Normalized Extremity Score (NES) was computed for each rating relative to the observed range of ratings for that specific movie.
- **Filtering:** Consistent with the main analysis, we included only reviewers with a history of at least 3 reviews to ensure the stability of the behavioral indices.
- **REI and RPI Computation:** The Reviewer Extremeness Index (REI) and Reviewer Polarity Index (RPI) were calculated for all valid users.

Figure 1 illustrates the distribution of reviewers in the REI-RPI space for the MovieLens dataset.

The comparative analysis between the Amazon Books and MovieLens datasets highlights key differences in reviewer behavior, showcasing the REI-RPI framework's ability to capture platform-specific dynamics.

The Amazon dataset (Figure 2 in the main manuscript) shows a clear concentration of reviewers in B1 (Moderate-Extreme Positive) and C1 (High-Extreme Positive) zones, indicating that Amazon users frequently provide extreme ratings, predominantly positive, typically in response to transactional experiences with products. The RPI respect to REI values suggest that while these reviews are extreme, they are overwhelmingly positive, reflecting the nature of consumer satisfaction in e-commerce environments.

In contrast, the MovieLens dataset (Figure 1) shows a different distribution. The figure indicates that most film reviewers cluster in the low-REI quadrants (A1, A2, A3), with a pronounced concentration in A1, where low extremeness is coupled with strong positive polarity. It also shows that, although MovieLens users tend to avoid extreme ratings, on the occasions when they do use them, their evaluations remain predominantly

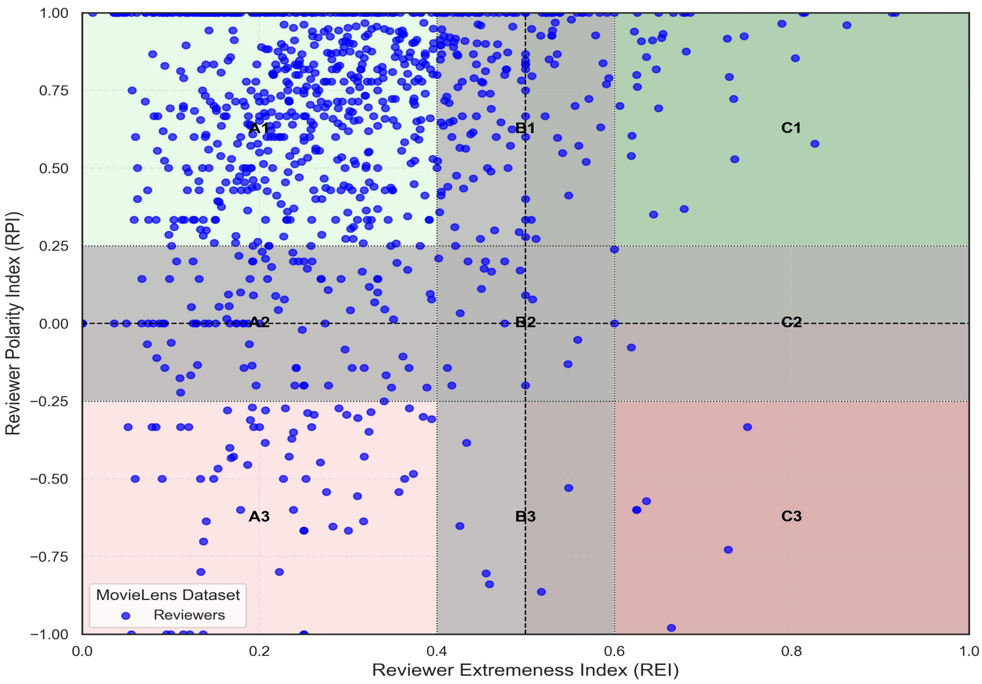

Figure 1: **REI—RPI in the MovieLens 100K Dataset.** The scatterplot displays the behavioral profiles of movie reviewers.

positive—an outcome that aligns with the more reflective and critically oriented style typical of film reviewing.

The REI-RPI framework effectively distinguishes between these two platforms: on Amazon, users are more inclined to use extreme ratings as signals of approval or disapproval, often leaning towards positive feedback. MovieLens users, on the other hand, engage in a more balanced and nuanced evaluation process, frequently using intermediate ratings to reflect their opinions.

This analysis underscores the flexibility and robustness of the REI-RPI framework in capturing different user behaviors across platforms, demonstrating its utility as a cross-domain profiling tool.
