# Supplementary material for: A two-dimensional framework for profiling online reviewer behavior
Source: PLoS One. 2026 Mar 25;21(3):e0344988. doi: 10.1371/journal.pone.0344988 (PMC13016354; doi:10.1371/journal.pone.0344988)
Supplement: S3 File — Jupyter notebook for REI-RPI framework analysis and to reproduce the results. (ZIP) [file pone.0344988.s003.zip › S3 Jupyter notebook.html]

S3 Jupyter notebook for REI-RPI adjust


# REI/RPI framework¶

- This file used in analysis is the the minimal derivative dataset uploaded on figshare (https://doi.org/10.6084/m9.figshare.30762815) required to reproduce the REI and RPI i
- The dataset includes cleaned observations and reviewers with at least three ratings.
- Only the variables strictly necessary for computing the REI and RPI measures are provided (Anonymized User\_id, Id (book Id), Title, review/score).
- The raw Amazon Book Reviews dataset is not included, as it originates from an external public repository (Kaggle – Mohamed Bakhet, 2022) and is not owned by the authors. Users wishing to access the full raw dataset may obtain it directly from the original source: https://www.kaggle.com/datasets/mohamedbakhet/amazon-books-reviews/data

In [1]:

```
%matplotlib inline
import pandas as pd
import xlrd
import re
import os
import numpy as np
from scipy import stats
import matplotlib.pyplot as plt
import seaborn as sns
from wordcloud import WordCloud, STOPWORDS

# Data Visualization
import plotly.graph_objs as go
import plotly.offline as py
import matplotlib.patches as mpatches
from matplotlib.lines import Line2D
import seaborn as sns
import numpy as np
from scipy.stats import spearmanr
#plt.style.use('fivethirtyeight')

## Network
import networkx as nx 
import pylab as plb 
from itertools import count 
from operator import itemgetter 
from networkx.drawing.nx_agraph import graphviz_layout 

import warnings
warnings.filterwarnings('ignore')
```

## 1. Exploring dataset¶

In [2]:

```
url_figshare = "https://doi.org/10.6084/m9.figshare.30762815"
ps = pd.read_csv("minimal_derivative_dataset_REI_RPI.csv")
ps.head(3)
```

Out[2]:

|  | Id | User\_id | Title | review/helpfulness | review/score |
| --- | --- | --- | --- | --- | --- |
| 0 | 1882931173 | d30b427b3093 | Its Only Art If Its Well Hung! | 7/7 | 4.0 |
| 1 | 0826414346 | 397a75604669 | Dr. Seuss: American Icon | 10/10 | 5.0 |
| 2 | 0826414346 | a69c4d66d363 | Dr. Seuss: American Icon | 10/11 | 5.0 |

In [3]:

```
#counting unique users
ps_uni=ps.User_id.unique()
ps_uni.shape
```

Out[3]:

```
(143107,)
```

In [4]:

```
# count the number of distinct books
ps_uni2=ps.Id.unique() # book title used to compute the number of distinct books
ps_uni2.shape
```

Out[4]:

```
(144565,)
```

## 3. Calculate NES (Normalized Extremity Score) for each review¶

In [5]:

```
# # Rename dataframe and create columns for NES calculation
df = ps.copy()
df.rename(columns={'Id': 'item_id', 'review/score': 'score'}, inplace=True)

# Calculate min and max scores per item
min_max_scores = df.groupby('item_id')['score'].agg(['min', 'max']).reset_index()
min_max_scores.rename(columns={'min': 'min_score_item', 'max': 'max_score_item'}, inplace=True)

# Joint the min and max scores back to the original dataframe
df = df.merge(min_max_scores, on='item_id', how='left')

# Calculate NES
def compute_nes(row):
    min_s = row['min_score_item']
    max_s = row['max_score_item']
    s_ij = row['score']
    
    if max_s == min_s:
        return 0
    else:
        return (2 * s_ij - (min_s + max_s)) / (max_s - min_s)

df['NES'] = df.apply(compute_nes, axis=1)
```

In [6]:

```
df.head(5)
```

Out[6]:

|  | item\_id | User\_id | Title | review/helpfulness | score | min\_score\_item | max\_score\_item | NES |
| --- | --- | --- | --- | --- | --- | --- | --- | --- |
| 0 | 1882931173 | d30b427b3093 | Its Only Art If Its Well Hung! | 7/7 | 4.0 | 4.0 | 4.0 | 0.0 |
| 1 | 0826414346 | 397a75604669 | Dr. Seuss: American Icon | 10/10 | 5.0 | 4.0 | 5.0 | 1.0 |
| 2 | 0826414346 | a69c4d66d363 | Dr. Seuss: American Icon | 10/11 | 5.0 | 4.0 | 5.0 | 1.0 |
| 3 | 0826414346 | b5394a56cdac | Dr. Seuss: American Icon | 7/7 | 4.0 | 4.0 | 5.0 | -1.0 |
| 4 | 0826414346 | 95a4d058a3f2 | Dr. Seuss: American Icon | 3/3 | 4.0 | 4.0 | 5.0 | -1.0 |

## 4. Calculate REI (Reviewer Extremeness Index)¶

We shift from the review to the reviewer.

In [7]:

```
# Create a boolean column for extreme reviews
df['is_extreme'] = df['NES'].abs() == 1

# Group by User_id to calculate REI
rei = df.groupby('User_id').agg(
    total_reviews=('NES', 'count'),
    extreme_reviews=('is_extreme', 'sum')
).reset_index()

rei['REI'] = rei['extreme_reviews'] / rei['total_reviews']
```

In [8]:

```
rei.head(4)
```

Out[8]:

|  | User\_id | total\_reviews | extreme\_reviews | REI |
| --- | --- | --- | --- | --- |
| 0 | 00003ebc0866 | 4 | 0 | 0.0 |
| 1 | 0000981b86a6 | 3 | 3 | 1.0 |
| 2 | 0000ae6f5405 | 3 | 0 | 0.0 |
| 3 | 0000bce09d57 | 3 | 3 | 1.0 |

## 5. Calculate RPI (Reviewer Polarity Index)¶

In [9]:

```
# Filter dataframe for extreme reviews only
extreme_df = df[df['NES'].abs() == 1].copy()

# Create boolean columns for positive and negative extreme reviews
extreme_df['is_positive_extreme'] = extreme_df['NES'] == 1
extreme_df['is_negative_extreme'] = extreme_df['NES'] == -1

# 3. Calculate n_pos and n_neg for each user
rpi_counts = extreme_df.groupby('User_id').agg(
    n_pos=('is_positive_extreme', 'sum'),
    n_neg=('is_negative_extreme', 'sum')
).reset_index()

# 4.Calculate RPI where possible (n_pos + n_neg > 0)
rpi_counts['total_extreme'] = rpi_counts['n_pos'] + rpi_counts['n_neg']
rpi_counts['RPI'] = (rpi_counts['n_pos'] - rpi_counts['n_neg']) / rpi_counts['total_extreme']

# 5. MERGE WITH REI
# Use a 'left' join starting from 'rei' to keep ALL users, including those who do not have extreme reviews.
rei_rpi = rei.merge(rpi_counts[['User_id', 'RPI', 'total_extreme']], on='User_id', how='left')

# 6. HANDLING MISSING VALUES 
# If a user is in 'rei' but not in 'rpi_counts', it means they have 0 extreme reviews.
# The merge will have produced NaN in the RPI column.
# We set it to 0 (Neutrality due to absence of extremism) to place them in zone A2.
rei_rpi['RPI'] = rei_rpi['RPI'].fillna(0)
rei_rpi['total_extreme'] = rei_rpi['total_extreme'].fillna(0)

# # 7. FINAL CHECK
print(f"Total number of reviewer in the original dataset: {len(rei)}")
print(f"Total number of reviewer in final dataset: {len(rei_rpi)}")
print(f"Reviewers retrieved (REI=0): {len(rei_rpi[rei_rpi['REI'] == 0])}")

rei_rpi.head()
```

```
Total number of reviewer in the original dataset: 143107
Total number of reviewer in final dataset: 143107
Reviewers retrieved (REI=0): 19284
```

Out[9]:

|  | User\_id | total\_reviews | extreme\_reviews | REI | RPI | total\_extreme |
| --- | --- | --- | --- | --- | --- | --- |
| 0 | 00003ebc0866 | 4 | 0 | 0.0 | 0.0 | 0.0 |
| 1 | 0000981b86a6 | 3 | 3 | 1.0 | 1.0 | 3.0 |
| 2 | 0000ae6f5405 | 3 | 0 | 0.0 | 0.0 | 0.0 |
| 3 | 0000bce09d57 | 3 | 3 | 1.0 | 1.0 | 3.0 |
| 4 | 0000f2c38c34 | 4 | 0 | 0.0 | 0.0 | 0.0 |

```
### 5.1 Graph analysis
```

### Calculate quantiles for REI and RPI¶

In [10]:

```
quartiles_RPI = rei_rpi['RPI'].quantile([0.25, 0.5, 0.75])
print(quartiles_RPI)
quartiles_REI = rei_rpi['REI'].quantile([0.25, 0.5, 0.75])
print(quartiles_REI)
```

```
0.25    0.0
0.50    1.0
0.75    1.0
Name: RPI, dtype: float64
0.25    0.333333
0.50    0.666667
0.75    1.000000
Name: REI, dtype: float64
```

### 5.2 Sub-analysis considering helpful ratio¶

In [11]:

```
# Split 'review/helpfulness' into two separate columns
df[['helpful_yes', 'helpful_total']] = df['review/helpfulness'].str.split('/', expand=True).astype(float)

# Calculate helpfulness ratio
df['helpful_ratio'] = df['helpful_yes'] / df['helpful_total']
```

In [12]:

```
# group by User_id to calculate helpfulness metrics
helpfulness_by_user = df.groupby('User_id').agg(
    total_helpful_votes=('helpful_yes', 'sum'),
    total_votes=('helpful_total', 'sum'),
    mean_helpful_ratio=('helpful_ratio', 'mean'),
    num_reviews_with_votes=('helpful_total', lambda x: (x > 0).sum())
).reset_index()
```

In [13]:

```
# Joint helpfulness metrics to rei_rpi dataframe
rei_rpi = rei_rpi.merge(helpfulness_by_user, on='User_id', how='left')

rei_rpi
```

Out[13]:

|  | User\_id | total\_reviews | extreme\_reviews | REI | RPI | total\_extreme | total\_helpful\_votes | total\_votes | mean\_helpful\_ratio | num\_reviews\_with\_votes |
| --- | --- | --- | --- | --- | --- | --- | --- | --- | --- | --- |
| 0 | 00003ebc0866 | 4 | 0 | 0.000000 | 0.0 | 0.0 | 1.0 | 1.0 | 1.0 | 1 |
| 1 | 0000981b86a6 | 3 | 3 | 1.000000 | 1.0 | 3.0 | 9.0 | 9.0 | 1.0 | 3 |
| 2 | 0000ae6f5405 | 3 | 0 | 0.000000 | 0.0 | 0.0 | 0.0 | 0.0 | NaN | 0 |
| 3 | 0000bce09d57 | 3 | 3 | 1.000000 | 1.0 | 3.0 | 0.0 | 0.0 | NaN | 0 |
| 4 | 0000f2c38c34 | 4 | 0 | 0.000000 | 0.0 | 0.0 | 0.0 | 4.0 | 0.0 | 4 |
| ... | ... | ... | ... | ... | ... | ... | ... | ... | ... | ... |
| 143102 | fffd4a6187f4 | 3 | 1 | 0.333333 | -1.0 | 1.0 | 1.0 | 1.0 | 1.0 | 1 |
| 143103 | fffd4bf58833 | 3 | 3 | 1.000000 | -1.0 | 3.0 | 6.0 | 30.0 | 0.2 | 3 |
| 143104 | fffe36f31939 | 4 | 4 | 1.000000 | -1.0 | 4.0 | 0.0 | 0.0 | NaN | 0 |
| 143105 | ffff2342f494 | 11 | 8 | 0.727273 | 1.0 | 8.0 | 12.0 | 12.0 | 1.0 | 8 |
| 143106 | fffff4ad5927 | 7 | 7 | 1.000000 | 1.0 | 7.0 | 7.0 | 7.0 | 1.0 | 7 |

143107 rows × 10 columns

In [14]:

```
# Set the helpfulness threshold
soglia_helpful = 0.8

# Create a color column: red if very helpful, otherwise gray
rei_rpi['color'] = np.where(
    rei_rpi['mean_helpful_ratio'] >= soglia_helpful,
    'purple',
    'lightgrey'
)

# Set the style
sns.set(style="white")

# Create figure
plt.figure(figsize=(10, 8))

# Color the four quadrants
plt.axvspan(0.5, 1, ymin=0.5, ymax=1.0, facecolor="#006400", alpha=0.3, zorder=0)   # Verde scuro - Quadrante I
plt.axvspan(0, 0.5, ymin=0.5, ymax=1.0, facecolor="#90ee90", alpha=0.2, zorder=0)    # Verde chiaro - Quadrante II
plt.axvspan(0, 0.5, ymin=0.0, ymax=0.5, facecolor="#f08080", alpha=0.2, zorder=0)    # Rosso chiaro - Quadrante III
plt.axvspan(0.5, 1, ymin=0.0, ymax=0.5, facecolor="#8b0000", alpha=0.3, zorder=0)    # Rosso scuro - Quadrante IV
plt.axhspan(-0.25, 0.25, facecolor="#b8b7b7", alpha=0.8, zorder=0)
plt.axvspan(0.4, 0.6, facecolor="#b8b7b7", alpha=0.8, zorder=0)


# Add the points, colored by helpfulness
for col in ['lightgrey', 'purple']:
    subset = rei_rpi[rei_rpi['color'] == col]
    plt.scatter(
        subset['REI'], subset['RPI'],
        s=30, alpha=0.5, color=col, label=f"Avg. helpfulness ratio ≥ {soglia_helpful}" if col == 'purple' else 'Others',
        zorder=1
    )

# Labels for the 9 zones A1–C3
rei_bounds = [0, 0.4, 0.6, 1.0]
rpi_bounds = [-1.0, -0.25, 0.25, 1.0]
zone_labels = [["A3", "B3", "C3"],
               ["A2", "B2", "C2"],
               ["A1", "B1", "C1"]]

# Place the zone labels at the center of each cell
for i in range(3):  #  RPI rows
    for j in range(3):  #  REI columns
        # Calculate cell center
        x_center = (rei_bounds[j] + rei_bounds[j+1]) / 2
        y_center = (rpi_bounds[i] + rpi_bounds[i+1]) / 2
        label = zone_labels[i][j]
        plt.text(x_center, y_center, label,
                 fontsize=12, color='black',
                 ha='center', va='center', fontweight='bold')


# Lines of the quadrants
plt.axhline(0, color='black', linestyle='--', linewidth=1)
plt.axvline(0.5, color='black', linestyle='--', linewidth=1)
plt.axhline(0.25, color='black', linestyle=':', linewidth=0.8)
plt.axhline(-0.25, color='black', linestyle=':', linewidth=0.8)
plt.axvline(0.4, color='black', linestyle=':', linewidth=0.8)
plt.axvline(0.6, color='black', linestyle=':', linewidth=0.8)

# Labels and title
plt.xlabel("Reviewer Extremeness Index (REI)", fontsize=14)
plt.ylabel("Reviewer Polarity Index (RPI)", fontsize=14)
#plt.title("Reviewer Behaviour: ERI vs DBI", fontsize=16)

# Limits
plt.xlim(0, 1)
plt.ylim(-1, 1)

# Grid
plt.grid(True, linestyle='--', alpha=0.3)

# Legend
plt.legend(title='Reviewers')

# Save figure
plt.tight_layout()
plt.savefig("fig2.tif", dpi=300, bbox_inches="tight", pad_inches=0.5)

plt.show()
```

### Tables with percentages of each zone¶

In [15]:

```
# define function to classify zones
def classify_zone(rei, rpi):
    if rei < 0.4:
        col = 'A'
    elif rei < 0.6:
        col = 'B'
    else:
        col = 'C'
    
    if rpi < -0.25:
        row = '3'
    elif rpi <= 0.25:
        row = '2'
    else:
        row = '1'
    
    return col + row

# Apply classification to each reviewer
rei_rpi['zone'] = rei_rpi.apply(lambda x: classify_zone(x['REI'], x['RPI']), axis=1)

# Count total reviewers per zone
zone_counts = rei_rpi['zone'].value_counts().reindex(
    ['A1', 'B1', 'C1', 'A2', 'B2', 'C2', 'A3', 'B3', 'C3'], fill_value=0
)

# Count reviewers with high helpfulness ratio per zone
purple_mask = rei_rpi['mean_helpful_ratio'] >= 0.8
purple_counts = rei_rpi[purple_mask]['zone'].value_counts().reindex(
    ['A1', 'B1', 'C1', 'A2', 'B2', 'C2', 'A3', 'B3', 'C3'], fill_value=0
)

# percentages
zone_percent_total = 100 * zone_counts / len(rei_rpi)
zone_percent_purple = 100 * purple_counts / zone_counts.replace(0, np.nan)  # evita divisione per zero

# Create summary table
zone_table = pd.DataFrame({
    'Zone': zone_counts.index,
    'Total Count': zone_counts.values,
    'Total %': zone_percent_total.round(2),
    'High Helpfulness Count (≥0.8)': purple_counts.values,
    'High Helpfulness % (per zone)': zone_percent_purple.round(2)
})

# Show the table sorted by Total %
zone_table.sort_values(by='Total %', ascending=False, inplace=True)
zone_table
```

Out[15]:

|  | Zone | Total Count | Total % | High Helpfulness Count (≥0.8) | High Helpfulness % (per zone) |
| --- | --- | --- | --- | --- | --- |
| zone |  |  |  |  |  |
| C1 | C1 | 72691 | 50.79 | 29525 | 40.62 |
| A2 | A2 | 20505 | 14.33 | 5961 | 29.07 |
| A1 | A1 | 13805 | 9.65 | 6093 | 44.14 |
| B1 | B1 | 10599 | 7.41 | 5036 | 47.51 |
| C3 | C3 | 10111 | 7.07 | 2038 | 20.16 |
| C2 | C2 | 5426 | 3.79 | 1862 | 34.32 |
| A3 | A3 | 5267 | 3.68 | 1873 | 35.56 |
| B2 | B2 | 2762 | 1.93 | 1172 | 42.43 |
| B3 | B3 | 1941 | 1.36 | 595 | 30.65 |

### Distribution of Highly Helpful Reviewers (Helpfulness ≥ 0.8) by Zone¶

In [16]:

```
# Filter only reviewers with helpfulness >= 0.8
purple_only = rei_rpi[rei_rpi['mean_helpful_ratio'] >= 0.8]

# Count how many reviewers are in each zone
purple_counts = purple_only['zone'].value_counts().reindex(
    ['A1', 'B1', 'C1', 'A2', 'B2', 'C2', 'A3', 'B3', 'C3'], fill_value=0
)

# Calculate the percentage relative to the total of "purple" reviewers
purple_distribution = 100 * purple_counts / len(purple_only)

# Create summary table
purple_table = pd.DataFrame({
    'Zone': purple_counts.index,
    'Count Helpfulness (≥0.8)': purple_counts.values,
    'Helpfulness %': purple_distribution.round(2)  # across all purple reviewers
})

# Sort by percentage in descending order
purple_table.sort_values(by='Helpfulness %', ascending=False, inplace=True)

# Display the table
purple_table
```

Out[16]:

|  | Zone | Count Helpfulness (≥0.8) | Helpfulness % |
| --- | --- | --- | --- |
| zone |  |  |  |
| C1 | C1 | 29525 | 54.52 |
| A1 | A1 | 6093 | 11.25 |
| A2 | A2 | 5961 | 11.01 |
| B1 | B1 | 5036 | 9.30 |
| C3 | C3 | 2038 | 3.76 |
| A3 | A3 | 1873 | 3.46 |
| C2 | C2 | 1862 | 3.44 |
| B2 | B2 | 1172 | 2.16 |
| B3 | B3 | 595 | 1.10 |

## B. Validation and robustness check¶

In [17]:

```
# Rename columns of cleaned reviews dataframe 
df_reviews = df.rename(columns={
    'User_id': 'reviewer_id',
    'score': 'rating',
    'review/time': 'review_time'
})

# reviewer-level index (REI, RPI, ecc.)
df_index = rei_rpi.rename(columns={'User_id': 'reviewer_id'})

# Ensure the extreme-flag column exists
if 'NES' in df_reviews.columns and 'is_extreme' not in df_reviews.columns:
    df_reviews['is_extreme'] = df_reviews['NES'].abs() == 1.0
```

In [18]:

```
# Reviewer-level variance (for convergent / discriminant validity)
def reviewer_variance(df: pd.DataFrame) -> pd.Series:
    """
    Compute the within-reviewer variance of the rating scores.
    This is used as a benchmark for convergent / discriminant validity.
    """
    return df.groupby('reviewer_id')['rating'].var(ddof=1).rename('var_rating')

def convergent_discriminant(df_reviews: pd.DataFrame, df_index: pd.DataFrame):
    """
    Convergent validity: association between REI and within-reviewer variance.
    Discriminant validity: association between RPI and within-reviewer variance.
    """
    var_rev = reviewer_variance(df_reviews)
    merged = df_index.merge(var_rev.reset_index(), on='reviewer_id', how='inner')

    # REI vs variance
    m1 = merged.dropna(subset=['REI', 'var_rating'])
    if len(m1) > 2:
        rho_rei, p_rei = spearmanr(m1['REI'], m1['var_rating'])
    else:
        rho_rei, p_rei = np.nan, np.nan

    # RPI vs variance
    m2 = merged.dropna(subset=['RPI', 'var_rating'])
    if len(m2) > 2:
        rho_rpi, p_rpi = spearmanr(m2['RPI'], m2['var_rating'])
    else:
        rho_rpi, p_rpi = np.nan, np.nan

    return {
        'n_for_convergent': int(len(m1)),
        'spearman_REI_vs_variance': {'rho': float(rho_rei), 'p': float(p_rei)},
        'n_for_discriminant': int(len(m2)),
        'spearman_RPI_vs_variance': {'rho': float(rho_rpi), 'p': float(p_rpi)},
    }
```

In [19]:

```
# Robustness to subsampling within reviewers
def compute_rei_rpi_from_reviews(df: pd.DataFrame) -> pd.DataFrame:
    """
    Compute REI and RPI from a review-level dataframe with NES and is_extreme.
    Assumes columns:
        - reviewer_id
        - NES
        - is_extreme (boolean)
    """
    # REI: share of extreme reviews per reviewer
    rei = df.groupby('reviewer_id')['is_extreme'].mean().rename('REI')

    # RPI: polarity among extreme reviews
    pos = (df['NES'] == 1.0).groupby(df['reviewer_id']).sum()
    neg = (df['NES'] == -1.0).groupby(df['reviewer_id']).sum()
    ext = pd.DataFrame({'pos': pos, 'neg': neg}).fillna(0)
    denom = ext['pos'] + ext['neg']

    rpi = pd.Series(np.nan, index=ext.index, name='RPI')
    mask = denom > 0
    rpi.loc[mask] = (ext.loc[mask, 'pos'] - ext.loc[mask, 'neg']) / denom[mask]

    idx = pd.concat([rei, rpi], axis=1).reset_index()
    return idx

def robustness_subsampling(df_reviews: pd.DataFrame, k: int = 3, drop_frac: float = 0.2, seed: int = 123):
    """
    Robustness check based on subsampling within reviewers:
    in each iteration, a fraction of reviews is dropped at random for each reviewer,
    and REI/RPI are recomputed and compared to the baseline.
    """
    idx_base = compute_rei_rpi_from_reviews(df_reviews)
    stats = []

    for it in range(k):
        def drop_some(sub):
            n = len(sub)
            if n <= 1:
                return sub
            n_drop = max(1, int(np.floor(n * drop_frac)))
            return sub.drop(sub.sample(n=n_drop, random_state=seed + it).index)

        sampled = df_reviews.groupby('reviewer_id', group_keys=False).apply(drop_some)
        idx_boot = compute_rei_rpi_from_reviews(sampled)

        merged = idx_base.merge(idx_boot, on='reviewer_id',
                                suffixes=('_base', f'_boot{it+1}'),
                                how='inner')

        d_rei = (merged['REI_base'] - merged[f'REI_boot{it+1}']).abs()
        d_rpi = (merged['RPI_base'] - merged[f'RPI_boot{it+1}']).abs()

        stats.append({
            'iteration': it + 1,
            'n_reviewers': int(len(merged)),
            'REI_MAE': float(d_rei.mean()),
            'REI_MedianAbsDiff': float(d_rei.median()),
            'RPI_MAE': float(np.nanmean(d_rpi)),
            'RPI_MedianAbsDiff': float(np.nanmedian(d_rpi)),
        })

    return {'subsampling_stats': stats}
```

In [20]:

```
# Temporal stability
def temporal_stability(df_reviews: pd.DataFrame, min_reviews: int = 10):
    """
    Temporal stability of REI and RPI:
    for reviewers with at least `min_reviews` and valid timestamps,
    split their review history into two halves and compare REI/RPI.
    """
    if 'review_time' not in df_reviews.columns:
        return {'note': 'review_time not available; skipped temporal stability.'}

    df = df_reviews[(df_reviews['review_time'].notna()) &
                    (df_reviews['review_time'] > 0)].copy()
    if df.empty:
        return {'note': 'no positive timestamps after filtering.'}

    df['review_time_dt'] = pd.to_datetime(df['review_time'], unit='s', errors='coerce')
    df = df.dropna(subset=['review_time_dt'])

    counts = df['reviewer_id'].value_counts()
    keep = set(counts[counts >= min_reviews].index)
    df_long = df[df['reviewer_id'].isin(keep)].copy()
    if df_long.empty:
        return {'note': f'no reviewers with >= {min_reviews} reviews.'}

    rows = []
    for rid, sub in df_long.sort_values('review_time_dt').groupby('reviewer_id'):
        t_med = sub['review_time_dt'].median()
        left = sub[sub['review_time_dt'] <= t_med]
        right = sub[sub['review_time_dt'] > t_med]

        if len(left) < 3 or len(right) < 3:
            continue

        rei_l = left['is_extreme'].mean()
        rei_r = right['is_extreme'].mean()

        def calc_rpi(part):
            pos = (part['NES'] == 1.0).sum()
            neg = (part['NES'] == -1.0).sum()
            den = pos + neg
            return np.nan if den == 0 else (pos - neg) / den

        rpi_l = calc_rpi(left)
        rpi_r = calc_rpi(right)

        rows.append([rid, rei_l, rpi_l, rei_r, rpi_r])

    if not rows:
        return {'note': 'insufficient data after temporal split.'}

    stab = pd.DataFrame(rows,
                        columns=['reviewer_id', 'REI_left', 'RPI_left',
                                 'REI_right', 'RPI_right'])

    d_rei = (stab['REI_left'] - stab['REI_right']).abs()
    d_rpi = (stab['RPI_left'] - stab['RPI_right']).abs()

    if len(stab) > 2:
        rho_rei, p_rei = spearmanr(stab['REI_left'], stab['REI_right'])
    else:
        rho_rei, p_rei = np.nan, np.nan

    mask = ~(stab['RPI_left'].isna() | stab['RPI_right'].isna())
    if mask.sum() > 2:
        rho_rpi, p_rpi = spearmanr(stab.loc[mask, 'RPI_left'],
                                   stab.loc[mask, 'RPI_right'])
    else:
        rho_rpi, p_rpi = np.nan, np.nan

    return {
        'n_reviewers': int(len(stab)),
        'REI_MAE': float(d_rei.mean()),
        'REI_MedianAbsDiff': float(d_rei.median()),
        'RPI_MAE': float(np.nanmean(d_rpi)),
        'RPI_MedianAbsDiff': float(np.nanmedian(d_rpi)),
        'Spearman_REI_left_vs_right': {'rho': float(rho_rei), 'p': float(p_rei)},
        'Spearman_RPI_left_vs_right': {'rho': float(rho_rpi), 'p': float(p_rpi)},
    }
```

In [21]:

```
# Run validation and robustness checks

cd_results   = convergent_discriminant(df_reviews, df_index)
subs_results = robustness_subsampling(df_reviews, k=3, drop_frac=0.2, seed=123)
stab_results = temporal_stability(df_reviews, min_reviews=10)
```

In [22]:

```
print(f"Total number of reviews used for validation: {len(df_reviews)}")
print(f"Total number of reviewers with REI/RPI: {df_index['reviewer_id'].nunique()}")
print("\nConvergent / discriminant validity:")
print(cd_results)
print("\nRobustness to subsampling:")
print(subs_results)
print("\nTemporal stability:")
print(stab_results)
```

```
Total number of reviews used for validation: 1113502
Total number of reviewers with REI/RPI: 143107

Convergent / discriminant validity:
{'n_for_convergent': 143107, 'spearman_REI_vs_variance': {'rho': -0.2670431587256831, 'p': 0.0}, 'n_for_discriminant': 143107, 'spearman_RPI_vs_variance': {'rho': -0.3273519532222676, 'p': 0.0}}

Robustness to subsampling:
{'subsampling_stats': [{'iteration': 1, 'n_reviewers': 143107, 'REI_MAE': 0.060312794179918326, 'REI_MedianAbsDiff': 0.005980066445182675, 'RPI_MAE': 0.06505219894008166, 'RPI_MedianAbsDiff': 0.0}, {'iteration': 2, 'n_reviewers': 143107, 'REI_MAE': 0.060277243964180625, 'REI_MedianAbsDiff': 0.00574712643678163, 'RPI_MAE': 0.06487655373307748, 'RPI_MedianAbsDiff': 0.0}, {'iteration': 3, 'n_reviewers': 143107, 'REI_MAE': 0.060248989512392326, 'REI_MedianAbsDiff': 0.0056022408963585235, 'RPI_MAE': 0.0643568286869031, 'RPI_MedianAbsDiff': 0.0}]}

Temporal stability:
{'note': 'review_time not available; skipped temporal stability.'}
```
